# Personalized Risk Assessment of Drug-Related Harm Is Associated with Health Outcomes

**DOI:** 10.1371/journal.pone.0079754

**Published:** 2013-11-06

**Authors:** Andrea A. Jones, Fidel Vila-Rodriguez, William J. Panenka, Olga Leonova, Verena Strehlau, Donna J. Lang, Allen E. Thornton, Hubert Wong, Alasdair M. Barr, Ric M. Procyshyn, Geoffrey N. Smith, Tari Buchanan, Mel Krajden, Michael Krausz, Julio S. Montaner, G. William MacEwan, David J. Nutt, William G. Honer

**Affiliations:** 1 Department of Psychiatry, University of British Columbia, Vancouver, Canada; 2 Department of Radiology, University of British Columbia, Vancouver, Canada; 3 Department of Psychology, Simon Fraser University, Burnaby, Canada; 4 School of Population and Public Health, University of British Columbia, Vancouver, Canada; 5 Department of Anesthesia, Pharmacology and Therapeutics, University of British Columbia, Vancouver, Canada; 6 Department of Pathology and Laboratory Medicine, University of British Columbia, Vancouver, Canada; 7 Department of Medicine, University of British Columbia, Vancouver, Canada; 8 Centre for Neuropsychopharmacology and Molecular Imaging, Division of Brain Sciences, Imperial College London, London, United Kingdom; Tulane University Medical School, United States of America

## Abstract

**Background:**

The Independent Scientific Committee on Drugs (ISCD) assigned quantitative scores for harm to 20 drugs. We hypothesized that a personalized, ISCD-based Composite Harm Score (CHS) would be associated with poor health outcomes in polysubstance users.

**Methods:**

A prospective community sample (n=293) of adults living in marginal housing was assessed for substance use. The CHS was calculated based on the ISCD index, and the personal substance use characteristics over four weeks. Regression models estimated the association between CHS and physical, psychological, and social health outcomes.

**Results:**

Polysubstance use was pervasive (95.8%), as was multimorbid illness (median 3, possible range 0–12). The median CHS was 2845 (interquartile range 1865–3977). Adjusting for age and sex, every 1000-unit CHS increase was associated with greater mortality (odds ratio [OR] 1.47, 95% confidence interval [CI] 1.07–2.01, *p* = 0.02), and persistent hepatitis C infection (OR 1.29, 95% CI 1.02–1.67, *p* = 0.04). The likelihood of substance-induced psychosis increased 1.39-fold (95% CI 1.13–1.67, *p* = 0.001). The amount spent on drugs increased 1.51-fold (1.40–1.62, *p* < 0.001) and the odds of having committed a crime increased 1.74-fold (1.46–2.10, *p* < 0.001). Multimorbid illness increased 1.43-fold (95% CI 1.26–1.63, *p* < 0.001).

**Conclusions:**

Greater CHS predicts poorer physical, psychological, and social health, and may be a useful quantitative, personalized measure of risk for drug-related harm.

## Introduction

 Misuse of tobacco, alcohol and illicit drugs continues to be a major threat to global public health [1-3]. Substance misuse contributes to a wide range of negative consequences to the health and psychosocial functioning of users [4]. Current, legislative approaches to mitigate illicit drug use purport to be based on severity of harm [5,6]. Seeking to inform drug legislation, an expert committee in the United Kingdom (UK), the Independent Scientific Committee on Drugs (ISCD) proposed a system for classifying 20 common drugs according to their potential harm [7]. The social and legal implications of quantifying risk of harm provoked significant debate, as well as corroborative research [8-10]. The possibility of applying the ISCD system to assess personal risk from using substances is unstudied. Evaluation is complicated by frequency of drug use, and concurrent use of multiple substances. Both factors are associated with poorer health outcomes [7,11-14], but create difficulties in measuring risk for harm related to multimorbid illnesses [1,4,15-18].

 We extended the ISCD system to individuals living in single room occupancy (SRO) hotels in Vancouver, Canada. Each tenant in an SRO lives in an 8 - 12 m2 room, possibly with a hotplate to prepare food. Toilet and shower facilities are shared by 10-15 tenants. This housing is frequently classified as “marginal” as there are often failures to meet one or more of the criteria for “acceptable” housing: 1) the dwelling must be in good repair according to residents (while many SROs are infested with pests or have fire safety concerns), 2) suitable housing includes assessment of the number of bedrooms relative to the composition of the household (toilet and shower facilities raise questions concerning suitability), 3) affordable housing costs less than 30% of before-tax income (rarely met) [19]. This type of housing has low barriers to tenancy, but high rates of tenants with multimorbid illness and substance use [14].

 We developed the Composite Harm Score (CHS) to build on the ISCD drug harm ranking system by incorporating personal substance use characteristics for the month preceding assessment to capture the overall potential harm to the individual. We assessed the strength of the association between the CHS and health outcomes to validate the CHS as a measure of drug-related harm that can be monitored over time. We hypothesized that the CHS would be associated with poorer physical, psychological, and social health. We performed regression analyses to examine the relationship between the CHS and infection, psychiatric diagnoses, psychosocial functioning, and total multimorbid illness burden at study entry, and mortality over a two-year period.

## Methods

### Study Design and Participants

 Participants were recruited from four single room occupancy (SRO) hotels in Vancouver, Canada. Persons were eligible for the study if they were SRO residents, able to communicate in English, and provided written informed consent. These were the only inclusion/exclusion criteria. The study was approved by the Ethics Board of the University of British Columbia. Participants received comprehensive baseline and monthly follow-up assessments (for up to 39 months) including a detailed review of substance use behavior, and measures relevant to physical, psychological, and social health [14].

### Substance Use Contributions to the Composite Harm Score

**Figure 1:**
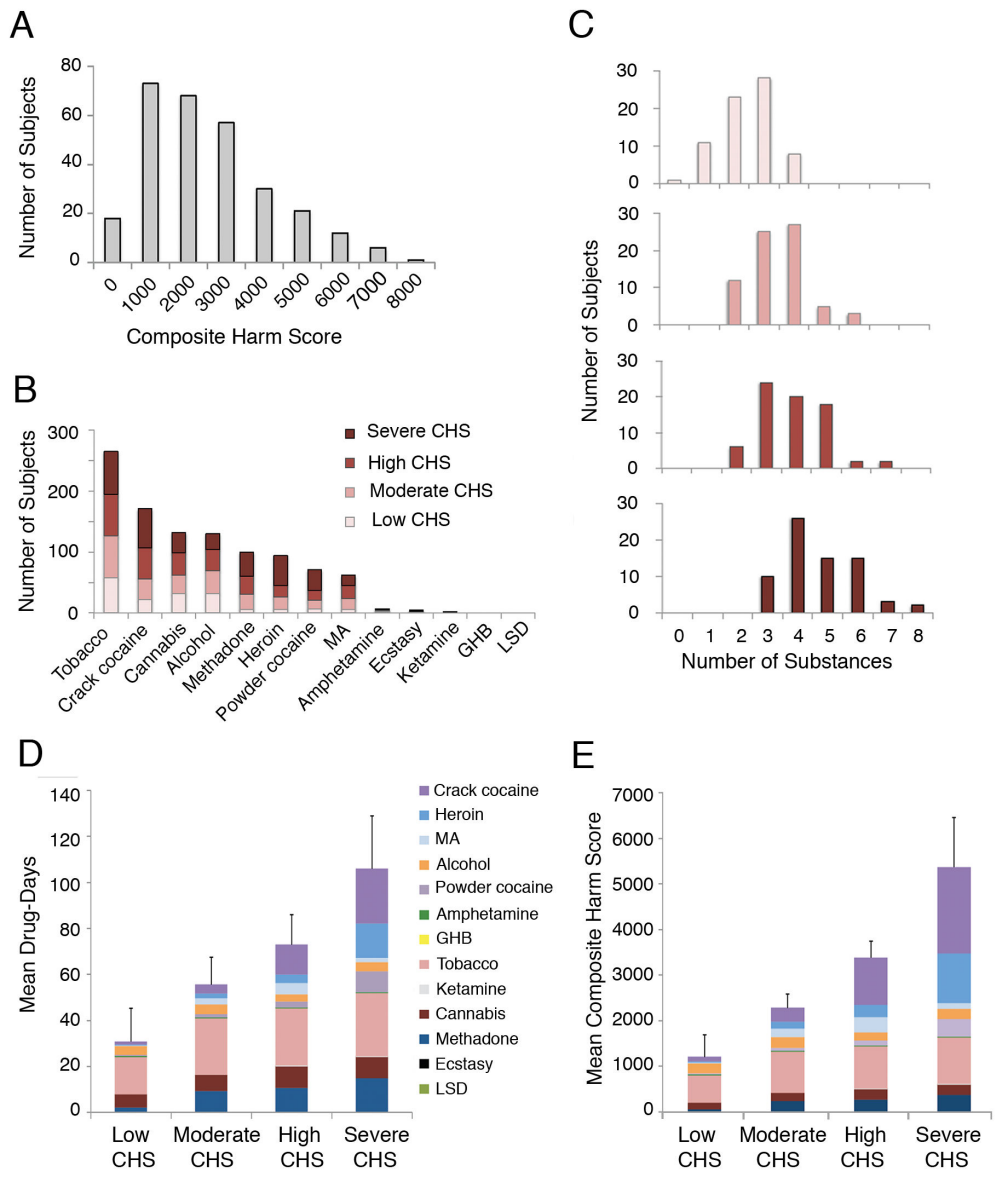
Composite harm scores. Panel A shows the distribution of Composite Harm Scores of the cohort from the first month of study. Panel B shows the prevalence of types of substance use during the first month of study across CHS quartile groups. MA: methamphetamine. Panel C shows the number of subjects in CHS quartile groups using multiple substances during the first month of study. Panel D shows the mean sum of the number of days using each substance during the first four weeks of the study for CHS quartile groups. Colors represent individual drugs, ordered from top to bottom in decreasing ISCD harm to user scores. Error bars indicate SD. Panel E shows the mean CHS for each CHS quartile group, colors indicating the contribution of harm from each substance (ISCD drug harm score * frequency) to the mean CHS value for the quartile group. Error bars indicate SD.

 Each of 20 substances scored by the ISCD multicriteria decision analysis model has a value from 0 to 100 based on harm to users [7]. The Composite Harm Score (CHS) applies this methodology to personalized assessment, incorporating the harm related to each type of drug used, as well as the monthly frequency of use, assessed with the Timeline Follow-Back method and a standardized Initial Substance Use Interview, both administered by a BA-level research assistant trained in Psychology [14,20]. Reports were verified with urine drug testing (kappa=0.66-0.70, see Table S1 in the File S1). Frequency of use was reported as the number of days using in the previous four weeks. The CHS calculation sums the drug-related harm to user across the 13 substances assessed here (Figure 1). The CHS is impacted by number and type of substances used, and by frequency of use by an individual participant. Composite Harm Score = ∑_1-13_ (ICDS Harm score x Frequency)

### Assessment of Health Outcomes

 Physical health outcomes focus on mortality, and infectious and neurological illnesses associated with stimulant and opioid exposure. For participants who died during the study, Coroner’s reports were requested, health care providers interviewed, and hospital records for the year prior to death were obtained. HIV/HCV exposure and current infection were tested using antibody detection, immunoblotting and qualitative polymerase chain reaction (qPCR) techniques. A screening neurological examination was carried out by a psychiatrist with the equivalent of US Board certification, and a magnetic resonance imaging (MRI) scan was obtained and reviewed by a neuroradiologist [14].

 Psychological health was assessed with a series of standardized interviews administered over two-to-three sessions by a research assistant, followed by a clinical interview, a mental status assessment by a psychiatrist, and cognitive testing supervised by a neuropsychologist [14]. We obtained records from participants with previous hospitalizations, from as long as 50 years previous. All available clinical information was reviewed by a research psychiatrist (WGH or FV-R) in a Best Estimate Clinical Evaluation and Diagnosis (BECED) process modified to make psychiatric diagnoses according to Diagnostic and Statistical Manual for Mental Disorders-Fourth Edition (DSM-IV) criteria [21,22].

 Social health and psychosocial functioning were evaluated for the month prior to baseline using the Role Functioning Scale and the Social and Occupational Functioning Assessment Scale (SOFAS) [22,23]. The Maudsley Addiction Profile questionnaire was administered to measure behaviors including self-reported criminal activity and employment [24]. Participants detailed the amount of money spent on non-prescribed drugs and alcohol in the previous four weeks. Participants reported any criminal activity in the month prior to baseline, with or without incarceration. Employment was reported as any days of formal employment in the last month. 

 To assess multimorbidity, we selected 12 illnesses (alcohol, stimulant or opioid dependence, psychosis, movement disorder, traumatic brain injury, seizures, cognitive impairment, brain infarction, active HIV, HCV or HBV infection) [14]. Each participant was assigned a multimorbidity score representing the sum of the illnesses present (range 0-12). If an illness such as brain infarction was not assessed due to contraindications for MRI, that illness was scored as absent.

### Statistical Analysis

 All participants with complete drug use information for the four weeks prior to study entry were included in analyses. Drug use characteristics (frequency and types of drugs) were compared between CHS quartiles using one-way analysis of variance (ANOVA). Post hoc comparisons using the Tukey honestly significant difference (HSD) test were used when relationships were statistically significant. Multiple regression models were constructed to identify associations between the CHS (calculated for the 4 weeks prior to study entry) and physical, psychological, and social health outcomes observed at baseline, adjusting for age and sex. These included: mortality (observed throughout the study period), persistent HCV infection, psychosis, depression, number of substance dependence diagnoses, psychosocial functioning, employment, drug spending, and criminal behavior. We describe effects with respect to a 1000-unit increase in CHS, approximately equivalent (as examples) to increasing the frequency of crack cocaine use from 15 days to 28 days per month, or to changing from methadone to heroin use (each at 21 days per month).

 Multiple logistic, linear, or Poisson-type regression models were used based on the distribution of the dependent variables, and satisfying model assumptions. For categorical measures, we used binary, ordinal, and multinomial logistic regression to estimate the adjusted odds ratio (OR) and 95% confidence interval (95% CI) associated with the CHS. Linear regression was used to estimate the adjusted effect coefficient and standard deviation (SD) of the CHS for continuous outcome variables. Quasi-Poisson regression was used to estimate the adjusted risk ratio (RR) and 95% CI associated with the CHS, accounting for over-dispersion of drug spending data. We calculated the predicted probabilities on the basis of the adjusted coefficients of the final regression models. Participants were excluded from an analysis when their data were missing. Significance was set at *p* < 0.05. We used JMP (version 9) and R (version 2.15.0) for data analysis.

## Results

### Participant Characteristics

 Between November 13, 2008 and July 31, 2011, all available SRO tenants were approached to participate; 293/406 (72.2%) met inclusion criteria and agreed to enroll. Of these, 288 participants had complete baseline substance use information and were included in analyses. Subjects were mostly middle-aged, white males though a substantial minority was of Aboriginal ancestry (Table 1). 

**Table 1:** Demographic measures and Composite Harm Score (n=288).

**Characteristic**	**Value**
**Age** - yr	
Median	44
Interquartile range	37–51
**Male sex** – no. of participants/total no. (%)	219/288 (76.0)
**Ethnicity** – no. of participants/total no. (%)	
White	170/288 (59.0)
Aboriginal	81/288 (28.1)
Black	7/288 (2.4)
Asian	1/288 (0.3)
Mixed/other	29/288 (10.1)
**Educational level** – no. of participants/total no. (%)	
Did not complete high school	163/288 (56.6)
Completed high school	125/288 (43.4)
Post-secondary degree, certificate or diploma	108/284 (38.0)
**Injected drugs in previous month** – no. of participants/total no. (%)	152/286 (53.1)
**Monthly income** (Canadian dollars)^a^	
Median	910
Interquartile range	662–1100
**Monthly disposable income** (Canadian dollars)^b^	
Median	535
Interquartile range	232–725
**Composite Harm Score**	
Median	2845
Interquartile range	1865–3977

a n=282

b n=282, monthly income less rent deducted at source ($375)

### Substance Use Contributions to the Composite Harm Score

 The CHS was calculated for 288 participants at baseline, and for 254 participants one month later, yielding an intraclass correlation coefficient of 0.74 (95% CI 0.68-0.79). The CHS distribution at baseline was positively skewed with a median of 2845 (interquartile range [IQR]: 1865-3977) and a mean (±SD) of 3077±1670 (Figure 1A). Drug use characteristics were stratified by CHS quartiles, referred to as low, moderate, high, and severe CHS (Figure 1B-C). The mean (±SD) values in each quartile group of 72 participants were: 1222±486, 2303±299, 3393±362, and 5390±1094, respectively. The mean CHS for women (3596±1588) was substantially greater than for men (2913±1666, *p* < 0.001), and women were more likely to be in the more severe quartiles of the CHS distribution (*p* = 0.004). 

 Inclusion in the CHS quartile groups could relate to number of drug types used, ISCD harm scores for specific drug types, and the number of days/month of use. Polysubstance use was pervasive: 276/288 (95.8%) participants used two or more substances during the previous month. The overall mean (±SD) number of substances used was 3.6±1.4. This value differed between CHS quartile groups (one-way ANOVA: *p* < 0.001) and was greatest in the severe CHS group (4.8±1.3, *p* < 0.001, Figure 1B). 

 Tobacco, crack cocaine, and cannabis were the most commonly used substances (Figure 1C). Across CHS quartiles, the prevalence of tobacco use ranged from 81.9 to 98.6%; cannabis use ranged from 38.9 to 52.8%, neither drug differed across quartiles. Crack cocaine use was significantly more prevalent in the severe CHS quartile (90.3% of members) compared with other quartiles (*p* < 0.001). 

 During the four weeks prior to baseline, more than half of the participants with severe CHS used crack cocaine daily, tobacco daily, methadone for >21 days, and heroin for 15 days or more. The mean number of days of using crack or powder cocaine, heroin, methadone, and tobacco differed across CHS quartile groups (all *p* < 0.001, Figure 1D). The most frequent use of heroin, crack or powder cocaine was observed in the severe CHS group (all *p* < 0.001). The least frequent use of methadone, and tobacco was seen in the low CHS quartile group (*p* = 0.002 and *p* < 0.001 respectively). The relative contribution of each drug to the mean CHS of each quartile is shown in Figure 1E.

### Health Outcome Measures

 Baseline physical, psychological and social health outcomes are presented in Table 2. At the time of analysis, during 543 person-years of observation, 14/288 (4.9%) of participants had died. The mean duration of followup for those who died (1.4 yr, range 0.3-3.1) did not differ from those still alive (2.0 yr, 0.0-3.2). The distribution of mortality (deaths/participants) according to CHS quartiles was: low CHS (0/72), moderate CHS (3/72), high CHS (6/72) and severe CHS (5/72). In addition to high rates of HCV exposure and persistent infection (Table 2), 52/279 (18.6%) of participants were HIV seropositive. Of five neurological illnesses assessed, at least one was present in 108/288 (37.5%) of participants (see Table S2 in the File S1 for additional details).

**Table 2:** Health outcome measures.

**Outcome**	**Value**
**Physical health – no. of participants/total no. (%)**	
Deceased	14/288 (4.9)
Hepatitis C virus exposure (seropositive)	197/279 (70.6)
Hepatitis C virus persistent infection	144/188 (76.6)
**Psychological health** – no. of participants/total no. (%)	
**Psychotic illness^a^**	136/288 (47.2)
Functional psychosis^b^	48/288 (16.7)
Psychosis not otherwise specified	37/288 (12.8)
Substance–induced psychosis	49/288 (17.0)
Psychosis due to a general medical condition^c^	2/288 (0.7)
Depressive illness^d^	56/288 (19.4)
**Number of substance dependence diagnoses**	
Median	3
Interquartile range	2–4
**Social health** – no. of participants/total no. (%)	
**Role functioning scale^e^**	
Median	12
Interquartile range	10–14
**Social and Occupational Functioning Assessment Scale^f^**	
Median	38
Interquartile range	31–45
**Committed a crime in past month**	95/283 (33.6)
Drug trafficking	60/283 (21.2)
Theft	31/283 (11.0)
**Any employment in past month**	42/281 (14.9)
**Drug spending in past month** (Canadian dollars)	
Median	350
Interquartile range	80–960
**Multimorbidity score** (number of illnesses, 0-12)	
Median	3
Interquartile range	2–4

a Information supporting diagnoses of a functional versus a substance-induced psychosis included ages at onset of first psychotic symptoms and of initial substance use, persistence and severity of psychotic symptoms and patterns of current substance use, as well as history of substance-independent psychotic episodes. In cases with a level of complexity including psychosis, stimulant dependence, and possible organic contribution from head injury or other medical illness, a diagnosis of Psychosis not otherwise specified was made according to DSM-IV criteria. Substance dependence diagnoses were informed by current patterns of substance use, evidence of tolerance and withdrawal, and degree of time and resources spent on obtaining and using the substance.

b Baseline: schizophrenia (n=21), schizoaffective (n=15), bipolar with psychosis (n=9), depression with psychosis (n=2), delusional disorder (n=1)

c Baseline: post-anoxic (n=1), interferon-related (n=1)

d Depression diagnoses included major depressive disorder, depression not otherwise specified, substance-induced depression or depression with psychosis according to DSM-IV criteria.

e n=284, Role Functioning Scale range: 0-28. Higher score indicates adequate functioning in the realms of work productivity, independent living and self-care, as well as positive immediate and extended social network relationships.

f n=287, SOFAS range: 0-100. Higher score indicates effective social and occupational functioning.

 Psychosis affected nearly half of the participants (Table 2). Nearly one-fifth suffered from a depressive illness. Substance dependence affected nearly all participants (283/288, 98.3%). 

 In terms of social health, standardized rating scales generated low mean scores, indicating clinically significant impairment (Table 2). Legal employment was infrequent; most participants relied on income from social assistance or long-term disability support. Participants spent much of their total legal disposable income on non-prescribed substances (median [IQR]: 67.5% [20.5-169.4%]). Drug spending often exceeded legal disposable income (115/279, 41.2%). Nearly one-third of the cohort committed at least one crime in the month prior to baseline assessment. Drug trafficking was most common, followed by theft. Subjects who spent more than 100% of their legal disposable income on substances were more likely to have committed a crime (*p* < 0.001). 

### Associations Between the CHS and Health Outcomes

**Figure 2:**
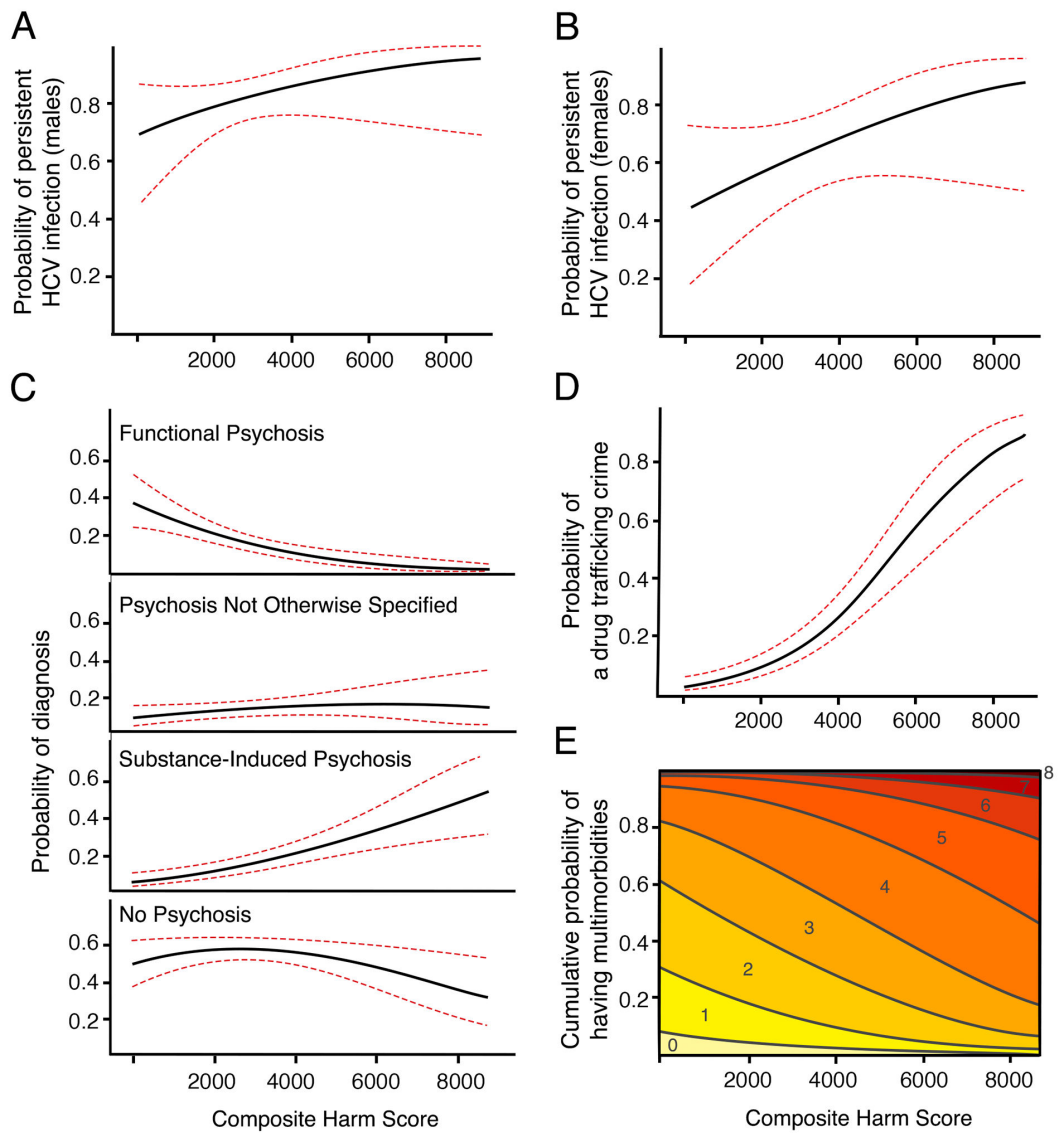
The effect of CHS on the probability of persistent HCV infection, specific psychosis diagnosis, drug trafficking criminal activity, and multimorbidity score. Estimated effect curve (black line) and 95% CI (red, dashed line) are presented for each plot. Panels A and B show the effect display of the influence of CHS on the probability of of persistent HCV infection in males (A) and females (B) adjusting for age. The vertical axis displays the probability of having an active HCV infection at the first serology screen. Panel C shows the effect display of the influence of CHS on psychosis diagnoses, controlling for age and sex. The vertical axis of each display is the probability of substance-induced psychosis, functional psychosis, PNOS, or no psychosis diagnosis, respectively. Panel D shows the effect display of the influence of CHS on the probability of engaging in drug trafficking, adjusting for age and sex. The vertical axis displays the probability of a drug trafficking crime being reported at the baseline assessment. Panel E shows the effect display of the association between CHS and the cumulative probability of having one or more of twelve multimorbid illnesses. Colored bands represent multimorbidity score, ranging from 0-8 in this display.

 Controlling for age and sex, the CHS predicted physical, psychological, and social health outcomes (Table 3). The adjusted effects of an increase in CHS on the probability of negative health outcomes are presented in effect displays (Figure 2) [25]. Higher CHS was associated with increased odds of death, and a higher likelihood of exposure to HCV. In a representative 41-year old female exposed to HCV, for an increase in CHS from 2500 to 3500 the probability of having persistent HCV infection increased from 0.604 (95% CI: 0.459-0.750) to 0.663 (95% CI: 0.534-0.792), while for a representative 45-year old male the probability increased from 0.807 (95% CI: 0.736-0.878) to 0.844 (95% CI: 0.777-0.910). 

**Table 3:** Regression analysis of association between composite harm score and health outcome measures^a^.

**Health outcome measure**	**n**	**Adjusted estimates of CHS effect (95% CI**)**^b^**	**p–value**
**Physical health**			
Mortality	288	1.47 (1.07–2.01)	0.016
Hepatitis C virus exposure	279	1.56 (1.28–1.92)	<0.001
Hepatitis C virus persistent infection	185	1.29 (1.02–1.67)	0.043
**Psychological health**			
**Psychotic illness**	286		
None (reference)		1.00	
Functional psychosis		0.73 (0.56–0.93)	0.014
Psychosis not otherwise specified		1.11 (0.89–1.38)	0.348
Substance–induced psychosis		1.39 (1.13–1.67)	0.001
Depressive illness	288	1.11 (0.93–1.32)	0.251
Substance dependence diagnoses	287	2.69 (2.29–3.19)	<0.001
**Social health**			
**Role functioning scale**	284	-0.02 (-0.27–0.23)	0.875
**SOFAS**	287	-0.44 (-1.22–0.34)	0.270
**Committed a crime in past month**	283	1.74 (1.46–2.10)	<0.001
Drug trafficking	283	1.97 (1.61–2.45)	<0.001
Theft	283	1.16 (0.93–1.44)	0.177
**Any employment in past month**	281	0.92 (0.73–1.13)	0.415
**Drug spending in past month**	283	1.51 (1.40–1.62)	<0.001
**Multimorbidity score** (0-12)	288	1.43 (1.26-1.63)	<0.001

^a^ Binary logistic regression was used to model the relationship between CHS and mortality, hepatitis C virus exposure, persistent hepatitis C Infection, depression, employment and committing any crime, drug trafficking or theft. Ordinal logistic regression was used to model the relationship between CHS and number of multimorbid illnesses and dependence diagnoses. Multinomial logistic regression was used to model the relationship between CHS and psychotic illness diagnosis. Linear regression was used to model the relationship between CHS and Role Functioning Score, and SOFAS. Quasi-Poisson regression was used to model the relationship between CHS and drug spending.

^b^ For binary, ordinal, and multinomial logistic regression models, adjusted odds ratios (95% CI) were reported for a 1000-unit increase in CHS, adjusting for age and sex. For linear regression models, adjusted effect coefficients (95% CI) for a 1000-unit increase in CHS, adjusting for age and sex. For quasi-Poisson regression models, the adjusted risk ratios (95% CI) were reported for a 1000-unit increase in CHS, adjusting for age and sex.

 The CHS was related to psychological health. A higher CHS was positively associated with a substance-induced psychosis diagnosis, and conversely, negatively associated with a functional psychosis diagnosis (Table 3). For an increase in CHS from 2500 to 3500 (in a 44-year old representative participant, used for this and subsequent descriptions where there was no sex-related difference), the probability of having a substance-induced psychosis diagnosis increased from 0.116 (95% CI: 0.071-0.161) to 0.156 (95% CI: 0.104-0.207) (Figure 2B). A higher CHS was also associated with having a greater number of substance dependence diagnoses (Table 3). The CHS was not associated with depressive illness. 

 The CHS also predicted social health outcomes. For an increase in CHS from 2500 to 3500, the probability of having committed a drug trafficking crime increased from 0.120 (95% CI: 0.072-0.167) to 0.211 (95%CI: 0.147-0.275) (Figure 2C). The CHS was positively associated with monthly spending on non-prescribed drugs and alcohol. However, the CHS was not significantly associated with measures from the Role Functioning Scale or SOFAS. 

 Greater CHS was highly associated with increased multimorbidity (Tables 3 and S2, and Figure 2D). We also examined the relationships between the CHS and health measures in the subsample of participants who injected drugs in the month prior to assessment (Table S3 in the File S1). Results were similar to the findings above, with diminished statistical significance likely due to the smaller sample size. 

## Discussion

 In participants with a range of types and frequencies of drug use, a personalized quantitative measure of drug-based harm predicted poorer physical, psychological, and social health. The high prevalence of tobacco, crack cocaine and cannabis use we observed is consistent with previous descriptions [26]. Women had higher CHS than men during the month prior to baseline assessment. This finding is supported by a report of greater use of crack cocaine by women than men living in this neighborhood, and increased engagement in unsafe practices such as crack pipe sharing [27,28].

 The CHS was associated with increased odds of mortality, consistent with the known risks of tobacco and alcohol use [16]. Cocaine, methamphetamine, and opioid use are also associated with increased mortality, most often attributed to overdose and HIV-AIDS [1,2,29,30]. Multiple substance use can increase risk of death due to acute toxicity [31-33]. Mortality associated with liver disease and HCV is increased in opioid users, with an additional contribution of concurrent alcohol dependence [34]. Here, the CHS was associated with increased odds of persistent HCV infection, consistent with other reports of the effects of opioid and alcohol abuse [32,35].

 The CHS was associated with increased probability of having a diagnosis of substance-induced psychosis, and a decreased probability of functional psychosis (including schizophrenia, schizoaffective disorder, bipolar disorder or depression with psychosis). Differentiating these disorders is challenging but important for prognostic purposes [36-38]. Cannabis, amphetamines and cocaine are associated with acute and chronic psychosis [1,39]. Not unexpectedly, the CHS was associated with having a greater number of substance dependence diagnoses. Having more concurrent substance dependence diagnoses was reported to be associated with more mental illness, antisocial behavior, and risk of overdose [40]. The CHS was not associated with a clinical diagnosis of depressive illness. Overall, depression was less prevalent that psychosis, and the absence of an association between the CHS and depression could be due to the aggregation for analysis of primary depression and substance-induced depression, necessary because of small numbers. As well, the prominent contribution of stimulant drugs to the CHS may obscure more specific relationships between individual drugs and depression. Finally, depression in people living in marginal housing may have more diverse origins than psychosis, including past history of trauma and perhaps a greater familial predisposition. 

 Substance dependence diminishes social and vocational function [32,41]. Not unexpectedly, the CHS was related to increased spending on drugs. This finding is reinforced by the strong relationship between the CHS and criminal activity, specifically drug trafficking. Lack of an association between the CHS and the SOFAS may be due to the narrow range of work activity carried out by members of the cohort - few reported any employment, and most depended on social benefits payments.

 Multimorbid illness is an important aspect of health outcomes, and in aging populations physical illness predisposes to mental illness [17,18]. In the present sample of middle-aged participants living in marginal housing, the CHS was associated with multimorbidity. Three of the twelve components of the multimorbidity score represent substance dependence. While the relationship with the CHS is not unexpected, the result supports the model of aggregating risk related to multiple substance exposure, with variable frequencies of use, as a measure of personalized liability for comorbid drug dependence, psychosis, and physical illness.

 There are limitations. First, substances such as mephedrone were not included in CHS analyses due to scarce use. Some substances such as hydromorphone are absent from the ISCD analysis [7]. Quantity of substance use, and the possibility of binge use, were not captured. The additive nature of the CHS may be a conservative approach, and does not address specific drug-drug interactions that may enhance harm. Additionally, acute versus chronic effects of substance use could not be parsed out in cross-sectional analyses. Finally, although the present sample was restricted in range for some outcome measures of interest, the range of the CHS was large. Further studies are needed to see if the CHS strategy is applicable in other clinical contexts.

 The CHS appears to provide meaningful information about risk to health from exposure to drugs, at least for socially marginalized individuals with a high prevalence of substance use and multimorbid illness, in a Canadian setting [14]. The approach rates harm related to crack cocaine as more severe than that from powder cocaine [7]. This may be of particular value in our sample, as both forms were used (crack more frequently) and different health consequences are related to the type of cocaine ingested [42,43]. The CHS was associated with physical, psychological, and social health outcomes, and may be more broadly applicable to other populations using substances. These individual-level findings support the value of the harm ranking system in setting priorities for public health interventions [7,10]. Reduction of the frequency or number of substances used may improve individuals’ health. No single measure can capture all the dimensions of drug exposure. The CHS offers an approach for clinicians to quantify the risk of drug-related harm, and may provide a measure for assessing the benefits from interventions. 

## Supporting Information

File S1 **Contains Tables S1-S3.**
Table S1. Validation of self-reported drug use by urine drug screen in baseline month (n=270). Table S2. Clinical measures of multimorbidity. Table S3. Regression analysis between Composite Harm Score and health outcome measures in injectors.
(DOC)
