# Supplementary material for: Personalized Risk Assessment of Drug-Related Harm Is Associated with Health Outcomes
Source: PLoS One. 2013 Nov 6;8(11):e79754. doi: 10.1371/journal.pone.0079754 (PMC3819243; doi:10.1371/journal.pone.0079754)
Supplement: File S1 — Contains Tables S1-S3. Table S1. Validation of self-reported drug use by urine drug screen in baseline month (n=270). Table S2. Clinical measures of multimorbidity. Table S3. Regression analysis between Composite Harm Score and health outcome measures in injectors. (DOC) [file pone.0079754.s001.doc]

**Supplemental Tables**

| **Table S1. Validation of self-reported drug use by urine drug screen in baseline month (n=270).** | | |
| --- | --- | --- |
| **Substance** | **Proportion observed** | **Kappa** |
| Methamphetamine | 87.6% | 0.66 |
| Cannabis | 82.8% | 0.66 |
| Cocaine (crack or powder) | 86.4% | 0.67 |
| Opiates | 85.9% | 0.70 |

| **Table S2. Clinical measures of multimorbidity.** |  | |
| --- | --- | --- |
| **Clinical characteristic** | | **Value** |
| **Substance dependence** – no. of participants/total no. (%) | |  |
| Stimulant (cocaine and/or methamphetamine) | | 236/288 (81.9%) |
| Opioid (heroin or other) | | 113/288 (39.2) |
| Alcohol dependence | | 55/288 (19.1) |
| **Mental illness** – no. of participants/total no. (%) | |  |
| Psychotic illness | | 136/288 (47.2) |
| **Neurological illness (active and/or current treatment)** – no. of participants/total no. (%) | | |
| Movement disorder^a^ | | 50/267 (18.7) |
| Any brain infarction on MRI | | 26/230 (11.3) |
| Traumatic brain injury (definite)^b^ | | 31/288 (10.8) |
| Seizures in past year and/or current treatment | | 26/287 (9.1) |
| Clinical cognitive impairment (DSM-IV) | | 19/288 (6.6) |
| **Infection** – no. of participants/total no. (%) | |  |
| anti-HIV positive | | 52/279 (18.6) |
| HCV viremia (HCV seropositive only) | | 144/188 (76.6) |
| HBV surface antigen positive | | 3/279 (1.1) |

^a^ Parkinsonism, dyskinesia or akathisia defined as a score of moderate or more on the Extrapyramidal Symptoms Rating Scale or the Barnes Akathisia Rating Scale

^b^ Evidence of previous traumatic brain injury on MRI (n=19); or history of traumatic brain injury (loss of consciousness ≥5 minutes or confusion ≥1 day) AND persistent symptoms referable to TBI (including seizures or organic personality disorder) (n=12)

| **Table S3. Regression analysis between Composite Harm Score and health outcome measures in injectors.^a^** | | | |
| --- | --- | --- | --- |
| **Health outcome measure** | **n** | **Adjusted estimates of CHS effect (95% CI)**† | **p–value** |
| **Physical health** |  |  |  |
| Mortality | 152 | 1.51 (0.98-2.41) | 0.063 |
| Hepatitis C virus exposure | 148 | 1.29 (0.90-2.00) | 0.201 |
| Hepatitis C virus persistent infection | 128 | 1.48 (1.10-2.11) | 0.017 |
| **Psychological health** |  |  |  |
| **Psychotic illness** | 151 |  |  |
| None (reference) |  | 1.00 |  |
| Functional psychosis |  | 0.73 (0.49–1.01) | 0.078 |
| Psychosis not otherwise specified |  | 1.04 (0.78–1.37) | 0.806 |
| Substance–induced psychosis |  | 1.24 (0.96–1.61) | 0.100 |
| **Depressive illness** | 152 | 1.17 (0.92–1.49) | 0.192 |
| **Substance dependence diagnoses** | 152 | 2.16 (1.76–2.67) | <0.001 |
| **Social health** |  |  |  |
| **Role functioning scale** | 150 | 0.14 (-0.14–0.42) | 0.331 |
| **SOFAS** | 151 | -0.40 (-1.40–0.59) | 0.425 |
| **Committed a crime in past month** | 150 | 1.60 (1.28–2.04) | <0.001 |
| Drug trafficking | 150 | 1.86 (1.46–2.44) | <0.001 |
| Theft | 150 | 1.13 (0.82–1.53) | 0.427 |
| **Any employment in past month** | 149 | 0.84 (0.60–1.15) | 0.305 |
| **Drug spending in past month** | 150 | 1.40 (1.28–1.54) | <0.001 |
| **Multimorbidity score** (0-12) | 152 | 1.34 (1.13-1.60) | 0.001 |

^a^ Binary logistic regression was used to model the relationship between CHS and mortality, hepatitis C virus exposure, persistent hepatitis C Infection, depression, employment and committing any crime, drug trafficking or theft. Ordinal logistic regression was used to model the relationship between CHS and number of multimorbid illnesses and dependence diagnoses. Multinomial logistic regression was used to model the relationship between CHS and psychotic illness diagnosis. Linear regression was used to model the relationship between CHS and Role Functioning Score, and SOFAS. Quasi-Poisson regression was used to model the relationship between CHS and drug spending.

^b^ For binary, ordinal, and multinomial logistic regression models, adjusted odds ratios (95% CI) were reported for a 1000-unit increase in CHS, adjusting for age and sex. For linear regression models, adjusted effect coefficients (95% CI) for a 1000-unit increase in CHS, adjusting for age and sex. For quasi-Poisson regression models, the adjusted risk ratios (95% CI) were reported for a 1000-unit increase in CHS, adjusting for age and sex.
